# Language-Related Skills in Bilingual Children With Specific Learning Disorders

**DOI:** 10.3389/fpsyg.2020.564047

**Published:** 2021-01-21

**Authors:** Anna Riva, Alessandro Musetti, Monica Bomba, Lorenzo Milani, Valentina Montrasi, Renata Nacinovich

**Affiliations:** ^1^Azienda Socio Sanitaria Territoriale (ASST) di Monza, Monza, Italy; ^2^Department of Medicine and Surgery, University of Milano Bicocca, Monza, Italy; ^3^Department of Humanities, Social Sciences and Cultural Industries, University of Parma, Parma, Emilia-Romagna, Italy

**Keywords:** specific learning disorder, bilingualism, metalinguistic abilities, children, language-related skills

## Abstract

**Purpose:** The purpose of this study is to better understand the characteristics of the language-related skills of bilingual children with specific learning disorders (SLD). The aim is achieved by analyzing language-related skills in a sample of bilingual (Italian plus another language) and Italian monolingual children, with and without SLD.

**Patients and methods:** A total of 72 minors aged between 9 and 11 were recruited and divided into four groups: 18 Italian monolingual children with SLD, 18 bilingual children with SLD, 18 Italian monolingual children without SLD, and 18 bilingual children without SLD. Each child underwent tests to evaluate different aspects of language skills: lexical and grammar, metalanguage and executive functions.

**Results:** With regard to lexical and grammatical skills, the conditions of SLD and bilingualism both impact naming in terms of total number of errors for words with low frequency of use, while the condition of SLD has an effect on semantic errors for words with low frequency of use. The condition of bilingualism impacts on the total errors for words with high frequency of use and on circumlocution-type errors for words with low frequency of use. There were significant effects of bilingualism and SLD on the metalinguistic test for understanding implicit meaning, and an impact of SLD on phonological awareness was also found.

**Conclusion:** The results suggest that both SLD and bilingualism have an effect on some lexical skills, in particular for words with low frequency of use. Both conditions, bilingualism and SLD, seem to impact on metalinguistic abilities that depend on lexical knowledge. These findings reinforce the importance of improving understanding of the neuropsychological profile of bilingual children with SLD.

## Introduction

In recent decades in Europe, due to an increase in the immigration rate, the number of bilingual children in schools has grown steadily (e.g., in Italy during the period 2001–2014, the rate quadrupled to 9%; Santagati and Ongini, 2015). Considering that estimates of the prevalence of specific learning disorders (SLD) in the developmental age indicate that approximately one student in each class (2.1% of all students) is affected and that the prevalence of developmental language disorders (DLD) is two students per class (as much as 7.5% of students), the need to improve our understanding of the neuropsychological characteristics of this population of bilingual children is clear (MIUR, 2015; Norbury et al., 2016; Raising Awareness of Developmental Language Disorder, 2020).

The aim of the current study is to investigate the characteristics of the language-related skills of a sample of bilingual (Italian and other language) and Italian native monolingual children with and without SLD, with a special focus on semantic-metalinguistic skills and linguistic measures of executive functions directly involved in the learning process. The novelty of this research derives from two main considerations. The first is that, despite great interest in studying the linguistic and neuropsychological profiles of children with isolated conditions of SLD or bilingualism, the co-presence of these characteristics in children has been little investigated (Kohnert et al., 2020). The second and no less important consideration is that many children with SLD have an unnoticed DLD, which commonly manifests itself in difficulty speaking and/or understanding a written text (Raising Awareness of Developmental Language Disorder, 2020).

The characteristics of the language-related skills of our participants (bilingual and monolingual, with and without SLD) are determined by studying a range of skills necessary for acquiring language and literacy. Ultimately, our study aims to better understand the characteristics of bilingual children with SLD. To clarify the focus of the present study, we start by providing a definition of the conditions of SLD and bilingualism and a review of the relevant literature.

### What Are Specific Learning Disorders?

SLDs are neurodevelopmental disorders that affect specific aspects of school learning (reading, understanding the meaning of what is read, spelling, writing, understanding numerical concepts/numerical facts/calculation, and performing mathematical reasoning), within a context of intellectual functioning that is otherwise appropriate to chronological age. Learning difficulties are not caused by other conditions, such as intellectual disability, vision or hearing problems, a neurological condition (e.g., pediatric stroke), adverse conditions such as economic or environmental disadvantage, lack of education, or difficulties in speaking/understanding the language. Learning disabilities refer to problems in one of three areas—reading, writing, and math—that are foundational to the ability to learn. These difficulties start during school age, leading to academic skills substantially below what is expected for the child's age, and causing problems at school and in everyday activities. According to the ability affected by the disorder (reading, writing, or math), SLDs are given specific names: dyslexia, dysorthography, or dyscalculia, respectively. According to the DSM-5, SLDs can be diagnosed only after formal education has started. A diagnosis is made through a combination of observation, interviews, and looking at family history and school reports (American Psychiatric Association, 2014).

This DSM-5 definition does not consider the presence of an undiagnosed DLD as closely linked with dyslexia; in fact, in cases of DLD, even if the child can read aloud accurately, there are often problems with understanding what is read (McArthur et al., 2000; Bishop and Snowling, 2004; Stothard et al., 2010).

DLD (previously called specific language impairment, SLI) is a common developmental disorder that constitutes the largest disability group in pre-school-aged children. Approximately 7% of the population is estimated to have DLD, but the condition has received relatively little research interest compared to other, less prevalent disorders, such as autism spectrum disorders (ASD) and attention deficit/hyperactivity disorder (ADHD) (Bialystok, 2011a). DLD often leads to dyslexia and may continue to restrict a person's social, academic, and occupational activities beyond adolescence and into adulthood (McArthur et al., 2000; Bishop and Snowling, 2004; Pennington and Bishop, 2009).

Over the past 20 years, research in the field of developmental neuropsychology has tried to identify the *core deficit* of SLD, and of dyslexia in particular. While the scientific community is generally in agreement in recognizing the role of poor phonological awareness and deficit in phonological processing, a consensus has recently been reached regarding the multifactorial origin of specific learning disorders (Vellutino et al., 2004; Snowling, 2019; Snowling et al., 2020).

For example, according to Pennington's multiple deficit model (MDM) of neurodevelopmental disorders, dyslexia is the outcome of multiple risks which accumulate toward a threshold for what is usually termed diagnosis (Pennington, 2006). The MDM is a multi-level framework spanning etiology (genes, environments, and gene–environment interplay), brain mechanisms, neuropsychology, and behavioral symptoms (McGrath et al., 2020). This model provides an explanation of comorbidity by positing that there are multiple possible risk factors for each neurodevelopmental disorder and that some of these risk factors are shared by comorbid disorders. For example, weaknesses in processing speed partially explain the comorbidity between dyslexia, dyscalculia, and ADHD, and weaknesses in oral language further contribute to the comorbidity of dyslexia and dyscalculia (McGrath et al., 2011; Peterson et al., 2017). Some cases of dyslexia in children (roughly 25%) can be adequately explained by single deficits (Pennington et al., 2012), even though multiple deficits contribute at the population level. However, the cognitive deficits identified are probabilistic predictors and cannot be considered as core. This is true even for the strong causal link between phonological awareness and reading abilities (Hulme and Snowling, 2013). In fact, Pennington's work suggests that approximately 50% of children with dyslexia do not have a phonological awareness deficit (Pennington et al., 2012). This finding is driving innovative research in dyslexia that moves beyond the so-called classic deficits and has important clinical implications for assessment and diagnosis (McGrath et al., 2020).

### What Is the Definition of Bilingualism?

Defining bilingualism has always been a complex challenge, but it has become more and more necessary over time, with, to date, more than half the global population speaking at least two different languages (Kohnert et al., 2020). Although the term *bilingual* is a relatively simple concept covering two languages, a definition that may be considered clear and exhaustive is not easily found. Some definitions may be better or more encompassing than others, but there is still no single correct definition. According to recent attempts, children who must learn and use two languages, either simultaneously from birth or sequentially in early childhood, can be classified as bilinguals, and bilingual individuals are those who “rely on two languages for meaningful interactions” (Kohnert et al., 2020). It is possible to classify bilingual children, on the basis of the age of L2 acquisition, into the following categories: (1) simultaneous bilingual children, who are exposed to two languages from birth or shortly after, and (2) consecutive bilingual children, who begin L2 acquisition after having already made significant progress in the L1.

In the first years of life, the learning of the L2 is almost comparable to that of the L1. Researchers disagree as to the critical age for L2 acquisition; some authors have identified the age of 7 as critical, but others have observed that children who begin learning a second language after the age of 3 experience a significant shift in how long it will take them to catch up with monolingual peers in speaking their new language (Fabbro, 1996; Weber-Fox and Neville, 1999; Perani et al., 2003; Schwartz, 2004; Fantauzzo and Roccella, 2008; Kovelman et al., 2008; Contento, 2011; Jasinska and Petitto, 2013; Bellocchi et al., 2016). Kovelman and colleagues distinguished between early bilinguals (EBs) and late bilinguals (LBs) according to the age of first bilingual exposure (lower or higher than age 3–4), which is when a bilingual child first begins to receive intensive, regular, and continued exposure to his/her new language (Kovelman et al., 2008).

### Bilingualism: Advantage or Disadvantage?

Over the years, there has been a progression from a negative view of bilingualism as potentially detrimental to cognitive development toward the current view that bilingual individuals present with greater cognitive resources than monolinguals, in particular in executive functions such as inhibition, task switching, and working memory (Carlson and Meltzoff, 2008; Bialystok, 2009, 2011a; Morales et al., 2013). A meta-analysis of 63 studies on the cognitive correlates of bilingualism revealed the largest mean effect sizes for attentional control (0.96), abstract and symbolic representation (0.57), and working memory (0.48) (Adesope et al., 2010). Furthermore, the bilingual advantage grows with increasing task complexity and demands on executive function. Concerning language development, research has shown that, although children most certainly have the ability to process dual language input and learn two languages, the rate of development in each language is somewhat slower than the rate for a single language—even for children. As a result, bilingual children lag slightly behind their monolingual peers in vocabulary and grammatical development when measured in each language separately (Gathercole and Thomas, 2009; Vagh et al., 2009; Bialystok et al., 2010; Marchman et al., 2010; Bialystok and Feng, 2011; Silvén et al., 2014; Hoff, 2015). The literature also indicates how variation in quantity and quality of input in each language affects the rate at which each is learned (Hoff and Core, 2013).

As a consequence, it should not be surprising that bilingual children, who receive on average less input in each language, take longer to learn each of their languages than monolingual peers take to learn just one (Hoff and Core, 2013). The size of the lag associated with bilingualism varies with age and the domain of language under consideration (Hoff and Core, 2015). As Bialystok has pointed out, the normal range of variation is wide in any case; thus, bilingual children can be delayed relative to monolingual children and still be within the normal range of variation (Bialystok, 2011b; Hoff et al., 2014). Research suggests that, in terms of grammatical development, bilingual children catch up with monolingual children in single language skills by the age of 9 or 10, although the differences in vocabulary size may be lifelong, because people potentially continue to learn new words throughout their lives (Bialystok et al., 2008). These differences in vocabulary size have been observed even when there are no differences in socio-economic status (SES) (Hoff and Core, 2013).

Empirical research has to some extent validated the assumption that competition may arise between two semantic units owing to parallel activation. Data converging on this idea show that bilinguals are slower on picture-naming tasks, produce fewer words in verbal fluency tasks, perform worse on lexical decision tasks, and experience much more difficulty with lexical access, despite similar receptive vocabulary scores (Ransdell and Fischler, 1987; Rosselli et al., 2000; Gollan and Acenas, 2004; Gollan et al., 2005; Bialystok, 2009; Yan and Nicoladis, 2009). Other studies have underlined that, although their rate of single language growth lags behind that of monolinguals, bilingual children's rate of total vocabulary growth is equal to or greater than that of their monolingual peers; moreover, a multilingual background generally leads to a greater ability to understand linguistic structures and their functioning intuitively (Bialystok, 2011b; Hoff et al., 2012; Core et al., 2013; Bosch and Ramon-Casas, 2014; Hoff, 2015). Other advantages of bilingualism reported in previous studies are improved metalinguistic awareness (the ability to recognize language as a system that can be manipulated and explored) as well as better memory, receptive abilities, visual-spatial skills, phonological skills, higher-level narrative skills, and even creativity (Diaz and Klingler, 1991; Baker, 2007; Oller et al., 2007; Friesen and Bialystok, 2012; Marian and Shook, 2012; Bialystok et al., 2014; Paradis and Kirova, 2014; Ribot and Hoff, 2014).

In terms of reading development, particularly decoding ability and oral and reading comprehension skills, some research has pointed out similar patterns in L1 and L2 learning paths in early and simultaneous bilingual children and a high degree of sensitivity to the systematic linguistic properties of their L2 (Genesee and Jared, 2008; Bellocchi et al., 2016). As outlined by August and Shanahan (2006), difficulties for L2 readers are linked to language proficiency and are more prevalent in reading comprehension than in decoding skills (August and Shanahan, 2006; Melby-Lervåg and Lervåg, 2013; Jeon and Yamashita, 2014). There is, however, a paucity of research that compares early and late bilinguals with both typically developing readers and struggling readers, such as children with dyslexia. In line with studies conducted on children learning English as an L2, Bonifacci and Tobia (2016) observed that both early and late bilingual children who acquire Italian as an L2 also reached monolingual-like levels of reading accuracy of words and non-words and non-word reading speed, but in word reading speed, both underperformed compared to typical monolingual readers (August and Shanahan, 2006; Bonifacci and Tobia, 2016). These findings seem to indicate that when lexical retrieval (and therefore linguistic competence) is involved, bilingual children are not as fast as their monolingual peers, despite being just as accurate. In this connection, Kovelman et al. (2008) found that early bilinguals exhibited monolingual performance in decoding tasks, and that both early and late bilinguals performed worse than monolingual children in reading comprehension tasks (Kovelman et al., 2008).

### Bilingualism and Specific Learning Disorders

Diagnosis of SLD in bilingual children is very complex. Bilingualism neither causes nor facilitates/aggravates a possible condition of dyslexia or of SLD in general (Crescentini et al., 2012). This is one of the two pivots on which our study is based. Since there is no causal relation between the two separate conditions (bilingualism and SLD), the potential synergic effects of bilingualism and SLD on language skills can be investigated. The second assumption for the present research consists in excluding incomplete second language acquisition or a condition of socio-cultural disadvantage as primary causes of educational or learning difficulties (World Health Organization, 1992). Once these conditions are excluded, we can be sure that we are observing only the characteristics of language-related skills and their interaction.

The present study builds on recent work conducted by our research group where we have shown that bilingual and monolingual children affected by SLD do not differ in cognitive level, measured using WISC-IV, a clinical tool administered individually to assess the cognitive abilities of children aged between 6 years 0 months and 16 years 11 months (Riva et al., 2017). Yet, in terms of cognitive profile, the bilingual children had gaps in the semantic-metalinguistic areas. With WISC-IV, five composite scores can be calculated to give a full-scale IQ representing the child's complex cognitive abilities, as well as five additional scores: a verbal comprehension index (VCI), a visual-spatial index (VSI), a fluid reasoning index (FRI), a working memory index (WMI), and a processing speed index (PSI) (Wechsler, 2003). The results of the previous study indicated that bilingual children with SLD show weakness in VCI and in WMI, especially in the subtest clusters measuring functions useful to learning a language (such as “word reasoning” and “Gf-VERBAL cluster”). Apart from this, and in line with previous studies, few or no differences were found in learning abilities (Murineddu, 2011). These results show that, even in the absence of differences at the cognitive level, bilingual children with SLD present greater difficulties in the semantic-metalinguistic areas than their monolingual peers. However, the previous study did not disambiguate whether this weakness in metalinguistic abilities could be ascribed to SLD, to bilingualism, or to the association of the two conditions.

## Materials and Methods

### Participants and Procedure

Seventy-two children aged between 9 and 11 years (mean = 10.393; standard deviation = 0.666) participated in the research: 18 bilingual children with SLD, 18 bilingual children without SLD, 18 Italian native monolingual children with SLD, and 18 Italian native monolingual children without SLD. The selected age range (9–11 years) allowed to us to carry out a comparison of language skills in general and to evaluate in particular those metalinguistic skills that are not developed physiologically before the age of 8.

In order to ensure that the bilingual children were comparable with the monolingual Italian children from a linguistic point of view, we selected only bilingual children who have lived in Italy for at least 5 years and who have been exposed to the Italian language for at least 40–60% of the time. In particular, the selected bilingual children were all born in Italy, with the exception of four who arrived in Italy during the first 4 years of life and have had at least 5 years of schooling in an Italian-language school. This criterion was necessary because, for a diagnosis of SLD, a bilingual child must have attended an Italian-language school for at least 5 years (Cummins, 2000). All bilingual minors selected had a medium-to-high academic performance. Of the 36 bilingual children participating in the study, 28 have both parents from foreign countries and 8 have one Italian parent. From birth, all the bilingual children have spoken a language other than Italian at home with their families. The types of bilinguals studied are heterogeneous: seven Spanish–Italian, seven Arabic–Italian, five Romanian–Italian, five Albanian–Italian, two Chinese–Italian, two English–Italian, two French–Italian, one Bengali–Italian, one German–Italian, one Finnish–Italian, one Bulgarian–Italian, one Greek–Italian, and one Moldovan–Italian.

Since the study aims to compare the language-related skills in the Italian language of 72 minors with and without SLD through oral and reading tests, subjects with isolated diagnoses of dyscalculia, or dysgraphia (or with the isolated coexistence of both diagnoses) were not included. This decision was made because these disorders could act as confounding factors in the interpretation of the results of the linguistic tests, as dysgraphia and dyscalculia alter functions that are not directly associated with the linguistic skills assessed by those tests. All children with an associated known diagnosis of SLD or with a history of speech delay/disorder were also excluded from the study. The monolingual and bilingual children without SLD were recruited from schools in northern Italy, and the monolingual and bilingual children with SLD were recruited from the outpatient neuropsychiatric public services of the Department of Child and Adolescent Mental Health, ASST Monza.

For all the participants, we calculated an SES value using the Hollingshead Four-Factor Index (Hollingshead, 1975). This choice was made in consideration of the evidence in literature that underlines the role of SES as a possible factor influencing the cognitive profile and language skills of bilingual children (Peal and Lambert, 1962; Cummins, 1976). Parents and participants were informed of the purpose of the study, and written informed consent for their participation was obtained. The research was reviewed by and received ethical approval from the Institutional Review Board of ASST Monza.

The socio-demographic characteristics of the sample are summarized in Table 1. One-way ANOVA was used to compare the four groups with regard to the parameters of age and Pearson's chi-square test was used to compare them with regard to gender, SES and SLD typology. The four groups were found to be comparable for age, gender, and SLD typology, although they differed for SES.

**Table 1:** Socio-demographic characteristics of the sample.

	**(a) SLD-monolingual (*N* = 18)**	**(b) SLD-bilingual (*N* = 18)**	**(c) NOSLD-monolingual (*N* = 18)**	**(d) NOSLD-bilingual (*N* = 18)**	***p***
Mean age in years (SD)	10.637 (0.158)	10.304 (0.172)	10.254 (0.186)	10.377 (0.157)	0.345
Female gender (%)	12 (66.6)	8 (44.4)	11 (61.1)	6 (33.3)	0.167
**Diagnosis (%)**					
Isolated dyslexia	1 (5)	2 (10)			
Dyslexia + dysgraphia and/or dyscalculia	1 (5)	0 (0)			0.931
Isolated dysorthography	3 (17)	3 (17)			
Dysorthography + dysgraphia and/or dyscalculia	5 (28)	5 (28)			
Dyslexia + dysorthography	3 (17)	3 (17)			
Dyslexia and dysorthography + dysgraphia and/or dyscalculia	5 (28)	5 (28)			
**SES (%)**					
<19.5	0 (0)	1 (5)	0 (0)	1 (5)	
20 < SES < 29.5	5 (28)	5 (28)	0 (0)	3 (17)	
30 < SES < 39.5	6 (33)	11 (62)	0 (0)	5 (28)	<0.01
40 < SES < 54.5	3 (17)	0 (0)	4 (22)	4 (22)	
>55	4 (22)	1 (5)	14 (78)	5 (28)	

*SES, socio-economic status; SD, standard deviation; SLD, specific learning disorder*.

### Instruments

#### Lexical and Grammatical Language Skills

##### Peabody Picture Vocabulary Test-Revised (PPVT-R)

The Italian version of the Peabody Picture Vocabulary Test, revised edition (PPVT-R) (Dunn and Dunn, 1981), measures receptive (auditory) Italian vocabulary in children aged 4–12 years and provides a quick estimate of their Italian verbal ability and scholastic aptitude. The PPVT-R consists of 175 stimulus words and 175 corresponding image plates. Each image plate contains four black-and-white drawings, one of which best represents the meaning of the corresponding stimulus word. There are also five training words and image plates. The final raw score is established when a child incorrectly identifies six of eight consecutive items. The raw score is then converted into the final score through the conversion tables attached to the test. Higher scores are associated with better performances. The reliability of the PPVT-R reported in the test manual is Cronbach's alpha = 0.88.

##### Naming Test

The naming test (Brizzolara, 2003) evaluates Italian-language expression by testing the ability of children aged between 4.6 and 10.8 years to understand and name images, and then analyzing the typology of the errors. The test consists of a book with 104 items divided into words with low and high frequency of use. By means of the attached conversion table, it is possible to calculate the *Z* score for each type of error: semantic, phonological, circumlocution, perceptive, and non-response. In this study, we focused on total numbers of errors for words with low and high frequency of use. When statistically significant differences in total errors were found, we also analyzed semantic errors and circumlocution errors.

##### Grammaticality Judgment

The subtest, included in the Battery for the Assessment of Language in Children Aged 4–12 (BVL_4-12) (Marini et al., 2015), evaluates children's ability to judge the grammatical correctness of 18 Italian sentences of varying length and syntactic complexity. Grammatical correctness is evaluated through adjective–noun agreement, subject–verb agreement, article–noun agreement, use of the third person pronoun, and affirmative–passive sentences. For each correct answer, the children received 1 point with a maximum score of 18 for each language. The internal consistency of the subtest reported in the test manual is Cronbach's alpha = 0.773 and test-retest reliability is 0.803.For the sake of simplicity, in this study the test is regarded as evaluating grammar, even if grammatical correctness is itself a metalinguistic competence.

#### Metalinguistic Abilities

The concept “metalinguistic” is not easily defined and has been used to qualify notions of awareness, ability, and task without distinguishing among them (Bialystok, 2001). Ramirez and colleagues defined metalinguistic awareness as the ability to distance oneself from the content of speech in order to reflect upon and manipulate the structure of language (Ramirez et al., 2013). Bialystok noted that metalinguistic awareness necessitates that “attention is actively focused on … the explicit properties of language” (Bialystok, 2001). In this study, we adopt a cognitive framework, originally proposed by Bialystok and colleagues, that outlines two components of metalinguistic awareness that are central to this definition: executive control and language analysis (Bialystok and Ryan, 1985; Friesen and Bialystok, 2012). Simply put, control denotes attention and monitoring processes, and linguistic analysis refers to formal language knowledge.

##### Comics

The comics test, from the Metalinguistic Understanding Assessment Tests (PVCM) battery (Rustioni et al., 2009), investigates, in the context of metalinguistic development, the comprehension skills of children from 8 to 11 years old and allows an accurate determination of their ability to decode an ambiguous message. The interpretative uncertainties elicited by the test determine in the individual the activation of some metalinguistic control systems necessary for the integration and processing of ambiguous messages. The test consists of 13 vignettes with comic characters and speech balloons in Italian. Children must examine the vignettes and indicate the statement that represents the correct answer among four options. The alternatives to the correct answer are built by playing on different meanings linked to their literal and/or metaphorical interpretation, or by referring to elements and actions concerning a partial aspect of the message or to some details of the images that must be integrated for correct understanding to occur. Confusing and misleading alternatives of a lexical, syntactic, phonological, literal, and/or absurd nature have been introduced in each item. For instance, a vignette might consist of the following:

A character, making their way through other people, exclaims: “My expert archaeologist eye will think of this.” The examiner invites the child to read the vignette carefully and then asks: What does ‘my expert archaeologist eye' mean?” Then the examiner continues to read the options aloud: “A: an archaeologist who sees well; B: an archaeologist who has no vision problems; C: an archaeologist who has a lot of experience; D: an archaeologist who experiments on the eyes.” The child is required to choose the correct answer.

The raw score is obtained from the sum of the right answers, corrected for the age of the subject. The reliability of the whole test reported in the test manual is Cronbach's alpha = 0.88 and for the “comics” subtest is 0.75.

##### Understanding Implicit Meaning

The Understanding of Implicit Meaning from APL Medea test (Lorusso, 2009) measures the pragmatic skills of understanding and using verbal language in children aged 5–14 years. In particular, the test evaluates specific skills related to the ability of children to make inferences on unexplained content. It consists of three brief stories in Italian in the form of dialogues. The examiner reads one story at a time to the child and, at the end of each reading, asks questions concerning the story. If the examiner notices that the child did not understand the story, he or she can repeat it several times before asking questions. It is important to use different vocal intonations to represent the different subjects of the story, in order to make it easier for the child to differentiate the various characters. There is no time limit for the administration of the test. The following is an example:

Marco said rising from his chair: “You know I don't like carrots!” [read by the examiner with a certain intonation]. “Don't worry, I can get you something else” [read by the examiner with a second intonation, e.g., with a formal tone; the sentence is supposed to be uttered by a waiter in a restaurant]. Luisa answered, giving him the car keys, “If you were going to do that, you could have stayed home!” [read by the examiner with a third vocal intonation]. After reading the text the examiner asks a series of questions to evaluate the child's ability to draw inferences from the story. For example: “How will Marco get home?” (correct answer: “by car”) or “Where does the scene take place?” (correct answer: “in a restaurant”).

For each response, the examiner can assign a score of 0 (totally incorrect), 0.5 (partially correct), or 1 (totally correct) according to the level of understanding shown by the child. The total score, obtained from the sum of the individual scores, is converted into Z scores using the attached conversion tables. The internal consistency of all the items of the test reported in the test manual is Cronbach's alpha = 0.922.

#### Linguistic Measures of Executive Functions

##### Spoonerism

The Spoonerism subtest, from the battery for the evaluation of metaphonological skills (CMF) (Marotta et al., 2008), is a test of analytical phonological awareness, an ability dependent on metalinguistic skills that do not develop spontaneously like oral language. Analytical phonological awareness is a very sensitive indicator of exposure to the coding rules of the alphabetical system, and it hardly develops in people who experience a delay in learning the written language. The difficulty lies in the demand for a considerable level of phonemic analysis and synthesis ability, as well as for a large commitment to working memory (a skill very frequently compromised in dyslexic subjects). The test consists of 15 items, each made up of two Italian words. The child is required to switch the initial phonemes of the two words to create two new words. If the child takes more than 1 min to answer an item, the test is stopped. The examiner cannot repeat the pairs of words. One of the pairs consists of *ponte* (bridge) and *fiume* (river), which become *fonte* (source) and *piume* (feathers). The examiner awards 2 points if both words are correct, 1 point if only one word is correct, and 0 points if both answers are incorrect. The total score is obtained by adding up the points obtained during the test. The test-retest reliability reported in the test manual is >.8 in all the scales.

##### Phonological Fluency

This test, from the Battery for the Evaluation of Language in Children from 4 to 12 years (BVL) (Marini et al., 2015), assesses the ability to access the lexicon through a phonological strategy. The examiner asks the child to produce as many Italian words as possible that begin with the phoneme “f” and the phoneme “s” in 1 min each. For each correct answer, the children received 1 point. The test-retest reliability of the subtest reported in the manual is Cronbach's alpha = 0.865.

##### Semantic Fluency

This test, again from the Battery for the Evaluation of Language in Children from 4 to 12 years (BVL) (Marini et al., 2015), assesses the ability of the child between 4 and 12 years of age to select target words belonging to certain semantic categories from their internal lexicon. The examiner asks the child to produce as many Italian words as possible in the categories of “animals” and “objects” in 1 min each. For each correct answer, the children received 1 point. The test-retest reliability of the subtest reported in the manual is Cronbach's alpha = 0.908.

##### Five Points

The test, from NEPSY II (Korkman et al., 2007; Stievano et al., 2013), investigates non-verbal executive functioning and, in particular, graphic figural fluency. The material consists of two A4 sheets with 40 square-shaped matrices in which five points are arranged. On the first sheet the five points are arranged symmetrically, while in the second sheet they are arranged asymmetrically. The child is presented with each sheet in turn and asked to connect two or more points with straight lines to produce different combinations in 1 min. The child is not allowed to repeat configurations, draw lines that do not connect points, or draw curved lines. The raw score is given by the total number of acceptable images on the two sheets, and the result is converted into Z scores using a conversion table. In the 7- to 12-year-olds, internal reliability coefficients reported in the manual are *r* = 0.80 or greater for all the subtest.

## Analysis

In the analysis, the continuous variables are expressed through the mean and SD of the corresponding distribution, and the categorical variables are expressed as absolute or percentage frequencies. For categorical variables, a descriptive analysis of the distributions was carried out by means of contingency tables, and the *p*-values of frequency comparisons were evaluated using Pearson's chi-square test and Fisher's exact test. Since SES differs in the four groups, Pearson's bivariate correlations were calculated to evaluate the impact of SES on performance in the linguistic tests. Two-way ANOVAs/ANCOVAs with Bonferroni correction were conducted with SLD and bilingualism as independent variables, and the scores on the different tasks as dependent variables, and SES as covariate, where appropriate. The level of significance was set at *p* < 0.05. Statistical analyses were performed using the SPSS 26.0 software package (IBM Corporation, Armonk, NY).

## Results

Since the four groups differ in SES (see Table 1), analyses were conducted to evaluate the impact of SES on linguistic skills. Statistically significant correlations were found between SES and Peabody [*r* (70) = 0.386, *p* < 0.01], the naming test's semantic error for words with high frequency of use parameter [*r* (70) = 0.253, *p* = 0.03], the comics test [*r* (70) = 0.366, *p* < 0.01], and the understanding of implicit meaning test [*r* (70) = 0.322, *p* < 0.01]. No significant correlations were found between SES and the other tests.

Tables 2–4 summarize the descriptive statistics and results of the two-way ANOVAs/ANCOVAs with bilingualism and SLD as independent variables, the scores of the different tasks as dependent variables, and SES as covariate when indicated. Regarding lexical and grammatical skills in particular, there was a significant impact of bilingualism [*F*(1, 68)13.648, *p* < 0.01] and SLD [*F*(1, 68)4.526, *p* = 0.037] on the total errors in the naming test for words with low frequency of use, and a significant impact of bilingualism [*F*(1, 68)5.192, *p* = 0.026] on the total errors in the same test for words with high frequency of use. Analysis of the error typology showed a significant effect of SLD [*F*(1, 68)10.852, *p* < 0.01] on semantic errors for words with low frequency of use and a significant effect of bilingualism [*F*(1, 68)11.262, *p* < 0.01] on circumlocution errors for words with low frequency of use. No other significant impacts of the two conditions were found on other naming subtests or on the Peabody and judgment of grammatical correctness test.

**Table 2:** Descriptive statistics in the four groups and results of 2 × 2 ANOVA with bilingualism and SLD as independent variables and results of lexical and grammatical skills as dependent variables.

	**SLD-monolingual mean (SD)**	**SLD-bilingual mean (SD)**	**NOSLD-monolingual mean (SD)**	**NOSLD-bilingual mean (SD)**	**2 × 2 ANOVA**		
						***F*(1, 68)**	***p***
Peabody	105.72 (10.64)	98.56 (7.43)	108.22 (11.28)	101.28 (9.94)	B*	3.069	0.084
					SLD*	0.028	0.867
					B × SLD*	0.198	0.658
Total errors for words with low frequency of use	−0.69 (0.86)	−1.64 (0.81)	−0.34 (1.02)	−1.07 (0.99)	B	13.648	<0.01
					SLD	4.526	0.037
					B × SLD	0.162	0.688
Total errors for words with high frequency of use	−0.58 (1.06)	−1.16 (0.76)	−0.40 (0.91)	−0.96 (0.84)	B	5.192	0.026
					SLD	0.420	0.519
					B × SLD	0.009	0.924
Semantic errors for words with low frequency of use	−1.86 (1.05)	−1.94 (1.05)	−0.89 (0.93)	−1.06 (0.98)	B	0.194	0.661
					SLD	10.852	<0.01
					B × SLD	0.039	0.845
Semantic errors for words with high frequency of use	−1.44 (1.15)	−1.39 (0.78)	−0.83 (0.70)	−1.11 (0.76)	B*	0.000	0.997
					SLD*	1.761	0.189
					B × SLD*	0.380	0.540
Circumlocution-like errors for words with low frequency of use	0.48 (0.72)	−0.47 (1.77)	0.39 (0.97)	−0.52 (1.27)	B	11.262	<0.01
					SLD	0.148	0.701
					B × SLD	0.021	0.886
Circumlocution-like errors for words with high frequency of use	−0.01 (1.23)	−0.51 (2.02)	−0.08 (1.15)	−0.48 (0.93)	B	1.917	0.171
					SLD	0.004	0.948
					B × SLD	0.023	0.881
Judgment of grammatical correctness	3.94 (2.6)	4.55 (2.97)	3.94 (1.79)	3.66 (2.02)	B	0.087	0.769
					SLD	0.619	0.434
					B × SLD	0.619	0.434

*M, mean; SD, standard deviation; B, bilingualism; SLD, specific learning disorder*.

* *Corrected for SES*.

**Table 3:** Descriptive statistics in the four groups and results of 2 × 2 ANOVA with bilingualism and SLD as independent variables and results for metalinguistic skills as dependent variables.

	**SLD-monolingual mean (SD)**	**SLD-bilingual mean (SD)**	**NOSLD-monolingual mean (SD)**	**NOSLD-bilingual mean (SD)**	**2 × 2 ANOVA**		
						***F*(1, 68)**	***p***
Comics	−0.45	−0.51	0.36	−0.14	B*	2.036	0158
	(1.32)	(0.75)	(0.63)	(0.82)	SLD*	0.248	0.620
					B × SLD*	0.532	0.468
Implicit meaning	0.21	−0.71	0.52	0.12	B*	7.957	<0.01
	(0.80)	(0.93)	(0.65)	(0.78)	SLD*	5.129	0.027
					B × SLD*	2.201	0.143

*M, mean; SD, standard deviation; B, bilingualism; SLD, specific learning disorder*.

* *Corrected for SES*.

**Table 4:** Descriptive statistics in the four groups and results of 2 × 2 ANOVA with bilingualism and SLD as independent variables and results for linguistic measures of executive functions as dependent variables.

	**SLD-monolingual mean (SD)**	**SLD-bilingual mean (SD)**	**NOSLD-monolingual mean (SD)**	**NOSLD-bilingual mean (SD)**	**2 × 2 ANOVA**		
						***F*(1, 68)**	***p***
Spoonerism	18.06	16.83	24.28	23.83	B	0.262	0.610
	(8.09)	(8.84)	(5.32)	(4.31)	SLD	16.504	<0.01
					B × SLD	0.057	0.812
Semantic fluency	1.67	1.78	1.83 )	1.72	B	0.000	1.000
	(0.48)	(0.42)	(0.38	(0.46)	SLD	0.286	0.595
					B × SLD	1.143	0.289
Phonemic fluency	4.00	4.00	4.39	4.44	B	0.006	0.939
	(1.78)	(1.64)	(1.33)	(1.29)	SLD	1.339	0.251
					B × SLD	0.006	0.939
Five points	10.05	10.72	10.17	10.67	B	2.012	0.161
	(2.58)	(2.52)	(2.53)	(3.09)	SLD	0.400	0.529
					B × SLD	0.010	0.921

*M, mean; SD, standard deviation; B, bilingualism; SLD, specific learning disorder*.

Regarding metalinguistic skills, a significant effect of bilingualism [*F*(1, 68)7.957, *p* < 0.01] and SLD [*F*(1, 68)5.129, *p* = 0.027], covariate for SES, on the test of understanding implicit meaning emerged, while no effects of the two conditions on the comic test were found. In terms of linguistic measures of executive functions, there was a significant effect of SLD [*F*(1, 68) 16.504, *p* < 0.01] on the Spoonerism test, but no other effects of the two conditions were found. No other effects of the two conditions on other linguistic measures of executive functions emerged.

## Discussion

In the present study we analyzed the characteristics of language-related skills in a sample of Italian native monolingual and bilingual children (Italian and another language) with and without SLD. In particular, we sought to evaluate the influence, if any, of a co-presence of bilingualism and SLD on these language skills, taking note of the relevance of undiagnosed DLD in children with SLD (McArthur et al., 2000; Bishop and Snowling, 2004). We chose instruments that evaluate different aspects of language skills: lexical and grammatical skills, and meta-language and executive functions.

To ensure that the sample was as homogeneous as possible and to minimize bias related to second language acquisition deficits, we recruited only bilingual children with at least 5 years of education in an Italian school and who have been exposed to the Italian language for at least 40–60% of the time. These criteria were adopted to reflect the finding that at least 5–7 years of education in a second-language school are necessary to obtain a good scholastic linguistic competence in the second language, even for children who have acquired a good knowledge at the oral level (Cummins, 2000). Since the groups differed in SES, the impact of this variable was controlled for in the analyses when necessary, given its known role as a factor potentially influencing the cognitive profile and the language skills of bilingual children (Peal and Lambert, 1962; Cummins, 1976; Bonifacci et al., 2020b).

For lexical features, the analyses revealed the impact of SLD on words with a low frequency of use, both in terms of total errors and in terms of semantic errors, as well as the impact of bilingualism on the storage of words in general (both frequently and rarely used) and on circumlocution-type errors for words of low frequency. Otherwise, no impact of either condition on receptive vocabulary measured with the Peabody test emerged. This weakness in the use and storage of less commonly used words in children with SLD is in line with previous research and with recent theories that SLD is multifactorial in origin, with difficulties in the storage and recovery of the lexicon and in meta-phonological skills among the etiological causes (Cornoldi, 2007). Moreover, the presence of SLD, which makes it difficult to acquire the reading of words with low frequency of use, may hinder the use of such words and therefore their inclusion in the child's vocabulary (Pennington, 2006; Peterson and Pennington, 2012). Nevertheless a limited expressive vocabulary and a difficulty in learning new words are also typical in DLD, so it may be supposed that both conditions coexist to some extent in our SLD sample (Alt and Spaulding, 2011). In terms of bilingualism, the present results on naming test indicate a weakness in general, both for frequently and rarely used words. However, the absence of a main effect of the Peabody seem to suggest that bilingualism, in this study, actually impact mainly on low frequency words. These results are only partially in line with previous findings of a general weakness in vocabulary storage in bilinguals (Ransdell and Fischler, 1987; Gollan and Acenas, 2004; Gollan et al., 2005; Bialystok, 2009; Yan and Nicoladis, 2009). The results regarding circumlocution may also be attributed to socio-cultural factors, since in some cultures it is more common to refer objects by their function than by their verbal labels (De Lamo White and Jin, 2011). No other differences were observed in the groups in the grammar task. This result appears to contradict the hypotheses, previous formulated, of a possible unrevealed DLD in our sample of children with SLD, since grammatical difficulties are common in children with DLD, whether they are monolingual or bilingual (Bonifacci et al., 2020a). The results are otherwise in line with previous research that suggests that bilingual children catch up with monolingual children in terms of grammatical development by the age of 9 or 10 (Bialystok et al., 2008).

An interesting and innovative aspect of this study is its focus on metalinguistic skills in children with a co-presence of bilingualism and SLD in light of previous research that suggests a weakness in metalanguage for these population (Riva et al., 2017). Our results identify a significant effect both of bilingualism and of SLD in one of the tests used to investigate metalinguistic skills, namely understanding of implicit meaning. This seems to contradict recent findings that bilingual children may have an advantage over their monolingual peers in the metalinguistic area (Baker, 2007; Friesen and Bialystok, 2012; Marian and Shook, 2012). Bental and Tirosh (2007) argued that because bilinguals know two different languages and two ways of writing the same words, they are able to understand symbolic representations before their peers, while Friesen and Bialystok (2012) explained the advantage of bilinguals in metalinguistic abilities in terms of a compensation mechanism for weaker linguistic skills (Baker, 2007; Friesen and Bialystok, 2012). Our findings can be explained by the fact that understanding implicit meaning is a metalinguistic skill influenced by lexical knowledge. Given the negative impact of bilingualism on some lexical skills, as described above, bilingual children may experience more difficulties with the test for the understanding of implicit meaning. On the other hand, the negative effect of SLD on the same metalinguistic skill, which is in line with previous findings, suggests that metalanguage is the most powerful ability in basic reading and that it may be compromised in children with SLD (Dong et al., 2020). These results, if confirmed in future studies, suggest that bilingual children with SLD may have difficulties with metalinguistic abilities in contexts that require higher levels of lexical competence.

Concerning linguistic measures of executive functions of language, we observed a significant impact of SLD, but not of bilingualism, on the Spoonerism test of phonological awareness. This result confirms that phonological awareness is a crucial ability in the development of reading, which is well known to be particularly deficient in children with SLD (Boets et al., 2010; Melby-Lervåg et al., 2012). Conversely, studies of the impact of bilingualism on this task are not univocal; although some have found evidence for a bilingual advantage, others have found evidence for a frailty in bilinguals in this respect (Chen et al., 2004; Hipfner-Boucher et al., 2014; Bonifacci et al., 2018). In the four groups in the present study, no other significant differences emerged in phonological and semantic fluency, which confirms the findings of some previous studies on SLD but contradicts others that noted a specific deficit in children with SLD (Bental and Tirosh, 2007; Mengisidou and Marshall, 2019). The present results are otherwise congruent with recent research on bilingualism (Giovannoli et al., 2020). Finally, no effect of either condition emerged in the graphic fluency task. This may be due to the fact that the task is a measure of linguistic executive functions that does not involve directly language, and is therefore easier to perform both for children with SLD and for bilinguals.

Although the present research presents several strengths, included the relatively novelty of the topic, still little investigated, it has also several limitations. These include sample size and heterogeneity of the bilingual sample due to L1 diversity, that does not let to control possible effects of L1 on the explored tasks. Another possible limitation is represented by the use of the naming test by Brizzolara et al., that is a test existing only in an Italian version, widely used in clinical practice, that lets to investigate not only total errors for low and high frequent words, but also different typologies of errors generally little explored as circumlocution errors, but whose psychometric properties are not published. Finally, the presence of between group differences in SES, although controlled in statistical analyses, must be considered a limitation because SES might influence linguistic skills and therefore some of the effects emerged in the present research could be partially related to SES rather than bilingualism or SLD.

## Conclusion

In this study, we analyzed the characteristics of the language-related skills of bilingual children with SLD. The results suggest that both bilingualism and SLD may have an impact on lexical skills, especially for low frequency words. We recommend that this weakness should be further investigated, since it could be also an indicator of undiagnosed DLD in both bilingual and monolingual children (Bonifacci et al., 2020a). The results also suggest that both conditions, bilingualism and SLD, determine a difficulty in metalinguistic abilities that depend on lexical knowledge. Our data, if confirmed in further and larger studies, could therefore improve understanding of the neuropsychological functioning of bilingual children with SLD and help to target ad hoc rehabilitation programs more effectively.

## Data Availability Statement

The raw data supporting the conclusions of this article will be made available by the authors, without undue reservation.

## Ethics Statement

The studies involving human participants were reviewed and approved by the Institutional Review board of Azienda Socio Sanitaria Territoriale (ASST) di Monza, Italy. Written informed consent to participate in this study was provided by the participants' legal guardian/next of kin.

## Author Contributions

AR, MB, VM, and RN developed the design of the study. LM processed the experimental data and AR, AM, and LM performed the analysis. All the authors contributed to the final version of the manuscript.

## Conflict of Interest

The authors declare that the research was conducted in the absence of any commercial or financial relationships that could be construed as a potential conflict of interest.
